# Myostatin gene promoter: structure, conservation and importance as a target for muscle modulation

**DOI:** 10.1186/s40104-019-0338-5

**Published:** 2019-04-23

**Authors:** Carla Vermeulen Carvalho Grade, Carolina Stefano Mantovani, Lúcia Elvira Alvares

**Affiliations:** 1 Universidade Federal da Integração Latino-Americana, UNILA, Instituto Latino-Americano de Ciências da Vida e da Natureza, Avenida Tarquínio Joslin dos Santos, 1000, Foz do Iguaçu, PR CEP 85870-901 Brazil; 2 Departamento de Bioquímica e Biologia Tecidual, Universidade Estadual de Campinas – UNICAMP, Rua Monteiro Lobato, 255, Campinas, SP CEP 13083-862 Brazil

**Keywords:** CAAT box, E-box, Gene promoter, Myogenesis, Myostatin, SNP, Transcription factors, TATA box

## Abstract

Myostatin (MSTN) is one of the key factors regulating myogenesis. Because of its role as a negative regulator of muscle mass deposition, much interest has been given to its protein and, in recent years, several studies have analysed *MSTN* gene regulation. This review discusses the *MSTN* gene promoter, focusing on its structure in several animal species, both vertebrate and invertebrate. We report the important binding sites considering their degree of phylogenetic conservation and roles they play in the promoter activity. Finally, we discuss recent studies focusing on *MSTN* gene regulation via promoter manipulation and the potential applications they have both in medicine and agriculture.

**Electronic supplementary material:**

The online version of this article (10.1186/s40104-019-0338-5) contains supplementary material, which is available to authorized users.

## Overview on myostatin gene

Myostatin (MSTN) is a member of the TGF-β superfamily of growth and differentiation factors which acts as a negative regulator of skeletal muscle mass deposition [1]. In mice, *Mstn* knockout leads to hyperplasia and hypertrophy of muscle fibers, resulting in a striking increase in skeletal muscle when compared to wildtype animals. This increased musculature is a result of the influence MSTN has on cell cycle control genes, in particular p21, leading myogenic progenitor cells to permanently withdraw from the cell cycle [2]. In other words, MSTN is a potent inhibitor of skeletal muscle progenitor cells proliferation which acts during animal development to ultimately establish skeletal muscle size after birth.

Noticeably, natural mutations in *MSTN* are associated to the double muscled phenotype in several animals, such as the Belgian Blue and Piedmontese breeds of cattle, dogs and sheep [3–6], indicating that the function of MSTN is evolutionarily conserved among these animals. A human child bearing a mutation in the *MSTN* gene and presenting increased musculature was also identified [7], which further increased interest in the study of this gene in the past years.

In addition to its role during skeletal muscle formation, MSTN also regulates the homeostasis of this tissue after birth. In fact, higher levels of MSTN protein are observed in the blood stream or muscle fibers of patients suffering from muscle loss or wasting processes, such as cachexia, muscular dystrophies and other muscle disorders [8–10]. Importantly, MSTN inhibition by specific antibodies seems to significantly increase muscle mass of dystrophic mice [11], which makes this molecule an important target for potential treatments of muscle wasting diseases. In fact, several approaches attempted to downregulate MSTN protein activity *in vivo*. For instance, a DNA vaccine was shown to increase body weight and skeletal muscle mass, without altering blood biochemistry or causing apparent side effects [12]. A neutralizing antibody and a dominant-negative receptor were also used to block MSTN protein function [13]. Thus, the development of technologies to modulate MSTN activity has great potential for application in both human health and in the development of livestock animals.

## *MSTN* gene promoter

Although most of the strategies to block or regulate MSTN activity have focused on its protein or receptor, it has already been reported that *MSTN* expression is also regulated at different levels. For instance, the microRNA miR-27b is able to attenuate *MSTN* expression in a posttranscriptional manner, supposedly through a putative recognition sequence in the 3′-untranslated region [14]. This indicates that other levels of regulation, including transcriptional regulation via elements such as a gene promoter, are also of importance.

Gene promoters are specific DNA sequences where RNA polymerase and basal transcription factors bind to drive gene expression [15]. Promoters are found at the 5′ region of the genes under their influence, and usually comprise a core promoter, which contains the information necessary for basal transcription machinery recognition and transcription start, and the proximal promoter, which is located upstream of the core promoter, and contains other critical sequences for transcriptional regulation, like tissue-specific transcription factor binding sites (TFBSs) [16].

In the past years, analyses of the *MSTN* gene promoter have shown that this regulatory element is conserved among animals and represents a potential target for the development of new strategies to modulate *MSTN* transcription. In this scenario, in the current review we will discuss the *MSTN* gene promoter structure and activity in different animal groups, as well as its conservation among them, in order to understand similarities and particularities. Lastly we will explore potential targeting strategies for medical and livestock production purposes.

## Human *MSTN* gene promoter

The first study detailing the structure and mechanisms involved in controlling *MSTN* promoter activity in human was based on a 3.3-kb segment of the 5′ regulatory region [17]. This region presents several potential binding sites for general and muscle-specific transcription factors, as summarized in Additional file 1. Among the most relevant are TATA and CAAT boxes, and 12 E-boxes, which are recognized and bound by the myogenic regulatory factors (MRFs), a family of transcription factors composed of MYOD, MYF5, MRF4 and MYOGENIN proteins, involved in all steps of skeletal myogenesis [18]. In fact, MYOD was already shown to be an upstream regulator of the *MSTN* gene [19], binding to the promoter region [20]. Also of myogenic importance, are two sites for myocyte enhancer factor 2 (MEF2), a critical transcription factor for myogenic differentiation [21].

Deletion analyses of the human 5′ regulatory region revealed that it contains a proximal segment of 327 bp containing essential elements for basal promoter activity, as well as for the responsiveness to glucocorticoids, which were shown to induce promoter activity, and increase endogenous MSTN, in a dose-dependent manner [17]. In addition, upstream of this proximal promoter are potential inhibitory elements (between bases 3322–2062 and 1447–1187) and putative enhancers (between 1187 and 529 and 529–327) of promoter activity, which may contribute to the regulation of *MSTN* gene expression in different contexts. In fact, the 5′ regulatory region was already shown to have higher activity in differentiated myotubes than in proliferating myoblasts [17], as was observed for *MSTN* mRNA [22] and endogenous MSTN [23]. This corroborates the role of *MSTN* promoter on gene expression in a myogenic context, which might be explored for medical applications in humans. Nonetheless, the majority of studies about the mechanisms controlling *MSTN* promoter activity were performed either *in vitro* or using model organisms, mainly the mouse, as will be discussed below.

## Murine *Mstn* gene promoter

**Fig. 1:**
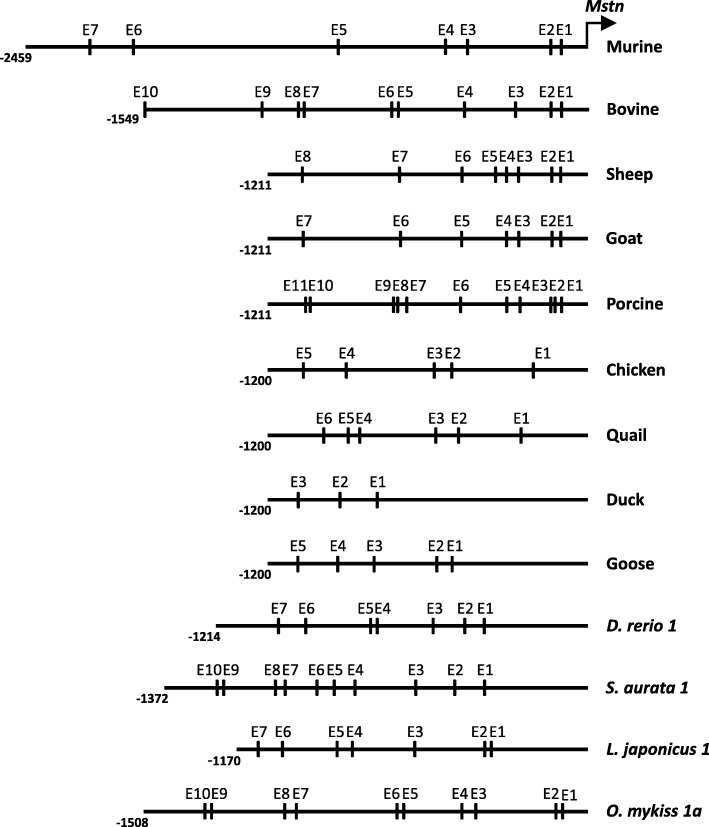
Schematic representation of E-boxes in the *Mstn*/*Mstn-1* promoter region of several animal species. E-boxes are numbered consecutively, as adapted from [24, 57, 73, 83]. Not all species addressed in this review were represented given the lack of information on the relative position of E-boxes. Same names and numbers of E-boxes do not necessarily implicate in homology

The study of 2.5 kb of the murine 5′ regulatory region showed the presence of several binding sites, such as for MEF2, C/EBP and CCAAT among others (see Additional file 1). Besides, Salerno et al. [24] showed the presence of seven E-boxes organized in four clusters (Fig. 1), which seem to work in an additive manner *in vitro*, indicating that these elements are also essential for *Mstn* expression, as in humans. The first five E-boxes, contained in the proximal 1 kb, were shown to be sufficient for obtaining the highest promoter activity *in vitro* and E-box number 5 appears to be crucial and capable of inducing maximal promoter activity by itself, being preferentially bound by the MRFs MYOD and MYF5, which were able to cause a 6-fold and 2-fold increase in the promoter activity, respectively [24]. Nonetheless, the most proximal fragment of 260 bp, including E-boxes 1–4, was able to induce gene activity, although at lower levels, and in silico analysis showed it displays 92% sequence identity with the human ortholog fragment [25]. Because of this homology, the murine *Mstn* promoter has been greatly studied under several conditions of human interest, as described below.

## Lessons from the murine *Mstn* gene promoter

### *Mstn* promoter role in the myogenic context

In the myogenic context, several activators of the *Mstn* gene promoter were already identified. For instance, insulin-like growth factor I (IGF-I) increases promoter activity most likely by an increase in cytoplasmic calcium [26], which leads to the activation of nuclear factor of activated T cells (NFAT) and CRE binding protein (CREB) [27]. Upon calcium increase, NFAT is dephosphorylated, accumulates in the nucleus, binds and activates gene promoter activity. In fact, several potential NFAT binding sites were identified in the mouse *Mstn* promoter [26]. A binding site for CREB was found in the *Mstn* promoter [25] and deletion constructs have indicated that CREB, together with NF-Y and MEIS1 binding sites, are essential for the basal control of *Mstn* transcription during early myogenesis [28]. Furthermore, IGF-I-mediated promoter activation was disrupted by cotransfection of CREB siRNA vectors, indicating a possible correlation between IGF-I signaling and CREB [27]. However, it is not clear whether these observed increases in activity represent a direct effect on the gene promoter, since no DNA-protein binding experiments were performed.

A few antagonists of *Mstn* promoter are also known. *Mstn* gene promoter is negatively regulated by the nuclear factor I/X (NFIX), which binds to specific sites and directly represses its activity in mouse myoblasts and *in vivo* [29]. By doing so, it controls the proper timing of satellite cell differentiation and muscle regeneration. Neuron-derived orphan receptor 1 (NOR-1) also represses *Mstn* promoter activity in mouse myoblasts, being possibly involved in the regulation of fatty acid use in skeletal muscle [30]. These findings might be useful for the development of strategies that aim at impairing *Mstn* transcription by targeting its promoter.

### *Mstn* promoter role in muscle atrophy

Glucocorticoids are known inducers of muscle atrophy and, in contrast to the observed for human and other animals (see below), the mouse promoter was only modestly induced by glucocorticoids *in vitro* [31]. This induction seems to be indirect and dependent on the upregulation of C/EBP, for which two putative binding sites were identified within 100 bp of the *Mstn* promoter. Curiously, these binding sites present complementary effects, since mutation of the first (− 100 bp) almost completely abolished basal promoter activity, but did not alter promoter responsiveness to C/EBP or GR (glucocorticoid receptor), while disruption of the second (− 50 bp) impaired promoter’s responsiveness to C/EBP while increasing basal activity [31]. In another scenario, C/EBP signalling was shown to cause a decrease in *Mstn* promoter activity in pluripotent C3H10T1/2 cells induced to differentiate into adipocytes [32], indicating a different biology function for these molecules in different cellular contexts.

In vivo assays, however, indicate that *Mstn* transcription is sensitive to glucocorticoids. When plasmids were transferred into the thigh muscle of mice treated with high doses of dexamethasone, there was an increase of 34% in *Mstn* promoter activity. This induction seems to be mediated by a GRE (glucocorticoid responsive element) motif, which, when mutated causes a 17% reduction of the promoter activity [33]. Together, these results indicate that glucocorticoids act on *Mstn* promoter in different manners according to the species, cellular context and through different pathways, both indirect and direct.

The murine *Mstn* promoter was also shown to be activated by FOXO1, which is a known regulator of genes involved in muscle atrophy, also increased during caloric restriction [34]. Five putative FOXO sites were identified across 1177 bp of the 5′ regulatory region and the most proximal one is conserved among human, cow, pig and goat. Deletion of all five sites drastically decreases FOXO-induced promoter activity, but not completely, indicating that this factor also acts in an indirect manner. Also conserved and located near the proximal FOXO site is a binding site for the SMAD proteins, which bind to the promoter and stimulate its activity, but independently from the FOXO site [34].

MSTN levels are also increased in hypothyroid rats [35]. Since *Mstn* promoter contains several regions responsive to hormones, it was suggested the alteration in promoter activity of hypothyroid rats induced by hormonal changes (in an indirect manner) might be involved in the muscle loss observed in such conditions.

### *Mstn* promoter role in sarcopenia

*Mstn* promoter was shown to be the involved in the development of sarcopenia, a muscle degenerative condition that accompanies hepatic cirrhosis [36]. It was shown that hyperammonemia caused by impaired hepatic function is associated to an increase in MSTN levels and a reduction in muscle mass. *In vitro* experiments showed that treatment of mouse myoblasts with ammonium acetate caused an increase in the binding of the nuclear factor kappa B – subunit 65 (NF-κB-p65) to the *Mstn* promoter, activating gene transcription. When NF-κB-p65 sites were deleted from the promoter region, as well as when NF-κB gene was silenced, this stimulation was abrogated [36].

### *Mstn* promoter role in other tissues

Besides muscle, *Mstn* is expressed in adipose, cardiac and liver tissues [1, 37], and its promoter was already shown to have important roles in them. For instance, although *Mstn* promoter activity is low in 3 T3-L1 mouse preadipocytes and after 4 days of differentiation, it was increased when cotransfected with expression constructs for the adipogenic transcription factor enhancer-binding protein-α (C/EBPα), proliferator-activated receptor-γ (PPARγ) and sterol regulatory element-binding protein 1c (SREBP-1c), indicating an upregulation of *Mstn* gene during conditions of adipose tissue growth. This indicates that *Mstn* promoter might have a role in the alterations observed in growth and metabolism of lean and fat tissues observed during obesity [38].

In rat cardiac myocytes, *Mstn* promoter activity was shown to be activated by angiotensin II (ANGII), a key molecule in cardiac remodeling and hypertrophy [39]. However, upon mutagenesis of the MEF2 binding sites located in the *Mstn* promoter, the increased activity induced by ANGII was abolished. This was also observed by the addition of MEF2 siRNA and a p38 MAP kinase inhibitor, suggesting that ANGII stimulation of *Mstn* promoter activity depends, at least in part, on p38 MAP kinase and MEF2, and may serve as a negative feedback mechanism to counteract the pathologic hypertrophy effects of ANGII [39].

## Bovine *MSTN* gene promoter

The study of 1.6 kb of the bovine upstream region showed it presents 79% of sequence identity with the human 5′ regulatory region and also bears many putative binding sites, including a CAAT site, three TATA boxes, besides a MEF2, which was shown to enhance promoter activity in vitro [19, 40] (see Additional file 1). Ten putative E-boxes are organized in three clusters within the bovine promoter (Fig. 1), with the two proximal clusters containing six E-boxes, which are sufficient to stimulate the same level of promoter activity observed for all ten E-boxes [19]. E-box number 6 appears to have a crucial role in regulation, and was indicated as the preferential binding site for the MRFs MYOD and MYF5, being able to compensate the loss of the other E-boxes. In accordance with the role of E-boxes in driving gene expression through MRFs, the promoter was demonstrated to be muscle-specific, presenting a weak activity when transfected into fibroblasts [19].

This bovine *MSTN* promoter was cloned upstream of the luciferase reporter gene, transfected into C2C12 cells and treated with increasing concentrations of wildtype MSTN protein. This resulted in a reduced promoter activity, indicating that *MSTN* promoter is under the negative control of MSTN protein [41]. However, when using the mutated MSTN protein from Piedmontese cattle, which presents a cysteine to tyrosine transition in the C-terminal active domain, promoter activity was not significantly affected. Furthermore, the negative feedback of *MSTN* promoter by MSTN protein was shown to be dependent on ActRIIB and ALK5 receptors, and SMAD7 protein. The gene promoter of SMAD7 was also shown to be activated by wildtype MSTN protein, but not by the mutated Piedmontese MSTN, while *MSTN* promoter activity was demonstrated to reduce upon SMAD7 expression. These observations indicate that the *MSTN* promoter is negatively auto-regulated by MSTN protein in a SMAD7 dependent manner, and the mutated MSTN protein observed in some double-muscled cattle breeds loses the ability to do so, leading to an increase in *MSTN* mRNA in such animals [41].

Single nucleotide polymorphisms (SNPs) were already detected in the bovine 5′ regulatory region of several breeds, such as Belgian Blue, Piedmontese, Limousine, Marchigiana, Black-and-White bulls, Holstein, Hanwoo, Jeju Black Cattle and Qinchuan cattle [40, 42–45]. The most relevant ones are summarized in Table 1. In some of them, a direct association between the promoter polymorphisms and the phenotype was found, which strengthens the potential use of *MSTN* promoter as a molecular marker, as well as a potential target for manipulation in improving economic traits of cattle breeds, as further discussed in the Potential Applications section. It is relevant to note that besides the effects on musculature, some SNPs also were associated with fat deposition [42], corroborating a role of MSTN in the balance between myoblasts and preadipocyte differentiation and muscle/fat deposition.

**Table 1:** Most relevant SNPs identified in the *MSTN* promoter region

Animal group	Breeds or species	SNP	Effect on *MSTN* promoter/gene	Phenotype changes	Ref.
Bovine	Marchigiana, Chianina, Romagnola, Piedmontese, Holstein Friesian, Italian Red Pied, Brown Swiss, Belgian Blue, Limousine	T/A (−371)	Introduction of a Pit1 (signal sequence TAAAT) and a WAP–US6 (signal sequence TTTAAA) binding motifs at − 372 and at − 374, respectively	Effect on muscularity only when associated with a mutation in the coding region (observed in Marchigiana)	[40, 44]
	Holstein (Dutch), Hanwoo and Jeju Black (Korean)		–	Effects on meat quality grade index and fat color index of backfat	[42]
	Marchigiana, Chianina, Romagnola, Piedmontese, Holstein Friesian, Italian Red Pied, Belgian Blue	G/C (− 805)	Introduction of an LBP-1 binding motif at − 806 (binding motif WCTRG)	–	[40, 44]
Porcine	Several commercial breeds	G/A (− 847); A/G (− 835)	Not significantly associated with *MSTN* expression levels; possible disruption of a MEF3 binding motif	Associated to growth, daily gain and meat quality traits	[53]
	Yorkshire	T/A (− 383)	–	Positive effects on birth weight and growth traits	[114]
	Large White, Landrace, Meishan, Wild boar, Piétrain, Yorkshire, Duroc	A/G (−447); G/A (− 435)	Disruption of a MEF3 binding motif (445–454); Differential expression of *Mstn* gene of muscle; likely to affect the recognition of E-box by MRFs; possible disruption of NFAT binding motif	Associated with body weight, higher muscle weight and percentage, decreased backfat thickness	[46, 49, 52]
	Meishan	T/A (−879)	Disruption of MSX1/MSX2 binding motif (873–885)	–	[46]
Sheep	*Ovis aries*	G/C (− 2449)	–	Connected to higher loin meat yields	[61]
		T/C (− 2379)	–	Associated with increased birthweight	
		C/T (− 1129) C/T (− 959)	–	–	[43]
		A/G (− 784)	–	–	
Horse	*Equus caballus*	T/C (−26)	–	Associated with heavy breeds, possible involvement in morphology traits	[64, 65]
		T/C (−156)	Affects TATA-3 binding motif		
Rabbit	*Oryctolagus cuniculus*	T/C (− 125)	–	–	[115]
		T/C (− 476)	–	Associated with increased liver weight and carcass weight; positive effect on growth	[116]
Chicken	*Gallus gallus*	A/T (− 214)	Possible disruption of FAST-1 binding motif	Higher body weights	[75]
Duck	*Anas platyrhynchos*	G/A (− 753)	–	Associated with breast meat percentages	[78]
		G/C (− 235)		Associated with abdominal fat percentages	
Fish	Atlantic salmon *(Salmo salar) mstn-1b*	C/T (− 1086)	–	Correlation with harvest weight, gutted weight, beheaded weight and fillet weight	[117]
	Flatfish spotted halibut *(Verasper variegatus) mstn-1*	T/C (− 355)	–	Correlation with growth traits in female individuals	[90]
Invertebrates	Noble scallop *(Chlamys nobilis)*	A/C (− 579)	–	Associated with growth traits	[118]
	Sea cucumber (*Apostichopus japonicus*)	A/G (− 779)	–	Associated with dry body weight	[97]
		T/C (− 437)	–		

A minus symbol (−) indicates unavailable information because it was not identified or tested. SNPs were considered as one when linkage disequilibrium was verified

## Porcine *MSTN* gene promoter

The analysis of 1.2 kb of the 5′ regulatory region of the porcine *MSTN* gene revealed a high degree of conservation with human (84.4%), mouse (71.6%), bovine (83.7%), sheep (82.2%) and goat (82.5%) [46, 47]. Despite the structural similarities with other species, the porcine promoter was shown to have a stronger activity in myoblasts than in myotubes, as opposed to the observed for human [17], indicating that the regulation of this gene promoter might be different for these species [32, 48]. Nonetheless, the porcine promoter was shown to be active in myoblasts but not in fibroblasts in culture, indicating there is still a certain degree of a tissue-specific regulation [48].

The alignment of these regions revealed the presence of two TATA boxes, which were conserved in all species, except in mouse. Based on mRNA sequences, it was possible to determine that the most distal TATA box (located between nucleotides − 71 and − 67 in pig) is responsible for transcription in pig and human, while the most proximal one is involved in the mouse transcription, resulting in a slightly smaller 5′ UTR in the rodent [47]. Several conserved binding sites for transcription factors were identified when aligning the pig and human 5′ regulatory region [32, 46, 47], as summarized in Additional file 1. As observed for mouse and bovine, the MEF2 factor was shown to bind and activate the promoter of the porcine *Mstn* gene [48].

Eleven E-boxes organized in clusters were identified in the porcine *MSTN* 5′ regulatory region and presented high degree of conservation with the ones observed in the bovine promoter [46] (see Fig. 1). Accordingly, the activity of the promoter was shown to increase in the presence of MYOD [32]. In fact, a length polymorphism was already described in some porcine breeds, in which an insertion of 386 bp containing four additional E-boxes was observed in some individuals [49].

Maternal nutrition was shown to have effects on *MSTN* promoter activity as was assessed in Meishan pigs, a slow-growing Chinese indigenous breed [50]. In the muscle of piglets at weaning (35 days), a low protein diet decreased nuclear C/EBPβ protein content as well as C/EBPβ binding to the promoter of *MSTN*, which possibly caused the downregulation in *MSTN* expression observed at this stage. On the other hand, at finishing stage (8 months of age), an upregulation of C/EBPβ binding to the *MSTN* promoter region as well as an increase in *MSTN* expression were reported, indicating that C/EBPβ is involved in both the immediate and the long-term effects of maternal dietary protein on offspring *MSTN* transcription [50]. Furthermore, in the muscles of commercial pigs tested at 28 days of age, low protein diet increased binding of FOXO3 and GRE to their putative binding sites in the *MSTN* promoter (− 3708, − 3535), indicating a possible role for these transcription factors in the activation of *MSTN* upon low protein maternal diet [51].

SNPs in the porcine *MSTN* promoter region were already reported by several authors [46, 49, 52, 53], and the most relevant are summarized in Table 1. Some of these single alterations possibly disrupt important binding sites, such as MEF3 [46, 52] and NFAT [49], a known activator of the *MSTN* promoter [26], leading to increased muscle deposition.

Interestingly, three of these SNPs are organized as four possible haplotypes: A (A^435^ -G^447^ -T^879^), B (G^435^ -A^447^ -T^879^), C (A^435^ -A^447^ -A^879^) and D (A^435^ -A^447^ -T^879^), in which haplotype D is the most ancestral, haplotypes B and C are derived from haplotype D, probably by artificial selection, and haplotype A originated from haplotype B [54]. The activity of the *MSTN* promoter harbouring haplotype A is significantly higher than the others, and its presence is associated with higher values of total meat weight and meat percentage in breeds such as Yorkshire, Landrace, Laiwu and Dapulian [52]. On the other hand, haplotype D presented the lowest activity, and was found only in wild boars [54]. Haplotype B, the dominant allele in Duroc pigs, was the second weakest, and was already reported to having a favourable effect on body weight along with daily gain in Yorkshire and Duroc pigs, as well as greater backfat thickness [49, 55]. Furthermore, haplotypes A and C were also active in CHO cells, suggesting that these polymorphisms also interact with transcription factors that are not muscle specific [56].

## Sheep *MSTN* gene promoter

The analysis of approximately 1.5 kb of the 5′ regulatory region of sheep revealed high degree of conservation with goat (98.1%), bovine (95.8%), porcine (86.9%), human (80.2%) and mouse (67.7%) [57]. Three different TATA boxes and one CAAT box were identified (respectively at − 139, − 163, − 523 and − 206 bp upstream ATG). TATA boxes 1, 2 and CAAT boxes are conserved both in position and sequence among sheep, goat, porcine and bovine, while TATA box 3 was not conserved in the porcine *MSTN* promoter [57]. Using three different softwares, Song et al. [58] concluded that the sheep *MSTN* core promoter is restricted to the region from 150 to 220 bp upstream of the start codon, in which they were able to identify the TATA box (comprised at 156–165 bp) and the CAAT box (at position 202–207 from the start codon).

Several muscle specific binding sites were also predicted in this region, as indicated in Additional file 1 [57, 58]. From these, some were conserved with other mammals, such as PRE, which was conserved between sheep and goat, MEF2, conserved among sheep, goat and bovine, and GRE, conserved in sheep, goat and porcine [57]. Binding sites for MEF2 and MTBF were shown to be important for the promoter activity, as well as the GRE, which seems to activate promoter activity via a glucocorticoid receptor-mediated pathway [59].

Within approximately 1.2 kb of the promoter, eight E-boxes were found (Fig. 1), from which seven (with the exception of E5) are conserved in position and sequence between sheep and goat and have correspondence with the porcine E-boxes as well. Six E-boxes (with exception of E4 and E5) are almost at the same positions as E1, E2, E3, E4, E5 and E7 of the bovine promoter [57]. Deletion experiments revealed that alterations in the E-boxes 3, 4, 5 and 7 had significant effects on promoter activity, being E-box 7 the most important of all sites. E-boxes 3, 4 and 5 are organized in a cluster, which is probably important for better stability of DNA-protein binding. E-boxes 3, 5 and 7 are possibly involved in the differentiation of myoblasts into myotubes, since a construct containing mutations in these three binding sites did not affect promoter activity, as opposed to the increase observed for the entire 1.2 kb 5′ regulatory region when cells were subjected to differentiation [60].

The whole 1.2 kb 5′ regulatory region of the sheep *MSTN* gene was cloned upstream of the reporter gene eGFP and was transfected into either mouse C2C12 myoblasts or sheep fibroblasts [60]. The results showed that the promoter was not able to direct reporter activity in fibroblasts, in agreement with the results observed for bovine [19] and porcine [48], indicating the muscle specificity of the *MSTN* promoter. Deletion experiments revealed that the initial 272 bp of the promoter region are already capable of driving reporter expression but the activity is the strongest with the complete 1.2 kb. Moreover, putative negative regulators were identified between 0.7 and 0.9 kb and between 0.3 and 0.4 kb. Additionally, increasing growth density of the C2C12 cells caused an inhibition of promoter activity as MSTN is known to inhibit cell proliferation [60].

Like in porcine and bovine, single nucleotide mutations were already reported in the sheep *MSTN* promoter sequence [43, 61], as summarized in Table 1.

## Goat *MSTN* gene promoter

Approximately 1 kb of the 5′ sequence of the *MSTN* gene from several breeds of Indian goat were analysed and shown to have 96.8% sequence identity with the sheep, 94.8% with cattle, 75.6% with pig, and > 60.3% with cat *MSTN* promoter. Three different TATA boxes and one CAAT box were identified, as well as six E-boxes (Fig. 1) and other binding sites (see Additional file 1) [62]. Besides, a 5-bp TTTTA deletion (position − 10 to − 6) was observed in several goat breeds, but absent in other species [62], and has a significant effect on body weight and size [63]. Furthermore, 16 SNPs were found among several Indian goat breeds [62], although no studies have yet investigated their roles on economically interesting traits.

## Equine *MSTN* gene promoter

A fragment of 670 bp of the equine *MSTN* 5′ regulatory region was analysed and shown to present 90% and 77% sequence identity with the pig and mouse promoter, respectively [64]. The alignment of the horse sequence with bovine, goat, human, mouse, pig and sheep indicated some conserved binding sites (summarized in Additional file 1), such as three TATA boxes, from which the second (TATA-2) was conserved among all organisms, while TATA-1 was not maintained in mouse and TATA-3 was not conserved in pig and mouse. Besides, four E-boxes were also identified, being E-4 the only one not conserved in human and mouse [64].

The search for SNPs in several horse breeds revealed the presence of two modifications in the promoter region (summarized in Table 1), which are organized in four possible haplotypes: 1 (T^26^, −T^156^), 2 (T^26^, −C^156^), 3 (C^26^, −T^156^) and 4 (C^26^, −C^156^) [64, 65]. According to frequency studies, haplotype 1 could be the most ancestral one, since it was present in all studied breeds [64].

A 227-bp long insertion was identified in the *MSTN* promoter region of Thoroughbred horses at − 146 position [66]. Further analysis identified this sequence as being a horse-specific repetitive DNA sequence element (SINE) known as equine repetitive element 1 (ERE-1), located at − 373/− 147 bp from the ATG site, and indicated it may disrupt or displace several important binding sites, such as E-boxes, FOXO, CCAAT and SMAD binding sites [66, 67]. Besides, the SINE contributes to the presence of extra putative binding sites for the Upstream Stimulator Factor (USF), a regulator of cell cycle and proliferation, for RAS-Responsive element binding protein 1 (RREB-1), involved in cell proliferation and differentiation, and for members of the NKX-homeodomain factor family [68]. A putative additional E-box and a TATA box-like motif were also identified [69]. Furthermore, the presence of the SINE was shown to displace the transcription starting site into the SINE insertion sequence, resulting in the production of an approximately 200 nucleotides longer mRNA [70]. Additionally, the insertion was shown to create a new CpG island, including a downstream segment at the insertion site, which might be target to epigenetic modifications [68]. To test if the SINE affected the strength of the *MSTN* promoter, the two variants (with and without the SINE) were cloned in a vector upstream to the GFP reporter gene and were transfected in human HeLa cells and horse fibroblast cells. In both cases, the presence of the SINE caused a reduction of reporter gene activity to almost undetectable levels, indicating that this insertion disrupts the *MSTN* promoter activity [71]. Accordingly, a luciferase system indicated that the presence of the SINE reduced *MSTN* promoter activity in both undifferentiated and differentiated C2C12 cells [70]. Noticeably, this SINE was also reported to correlate with aptitude and racing performance, since it possesses a significant association with muscle fibre type proportions in Thoroughbred and Quarter Horses, resulting in higher proportion of type 2B fibres, the fastest contracting and largest fibres, and lower proportion of type 1 fibres, slower contracting, smaller fibres [67, 72]. However, the SINE was not significantly associated with muscle fibre diameter, although fibre number was not verified.

## Rabbit *MSTN* gene promoter

Although the 5′ regulatory region of the rabbit *MSTN* gene has not been extensively studied, some authors identified the occurrence of SNPs in this region, as summarized in Table 1.

## *MSTN* gene promoter of birds

A 1.2-kb fragment of *MSTN* 5′ regulatory region was studied in birds revealing the presence of several E-boxes, which are conserved in number and location among chicken and quail [73] (Fig. 1). Other study showed that within 2.3 kb of *MSTN* 5′ regulatory region from Wengshang Luhua chicken sites for MEF2 and GRE are found, besides 13 E-boxes [74]. Natural occurring mutations in E-boxes 3 or 4 were shown to be responsible for significant decrease in the promoter activity *in vitro*, but only modifications in E-box 3 depicted reduction in vivo, showing that other regulatory mechanisms present in the organisms and not in the cell culture are involved in the promoter regulation [74].

*MSTN* promoter of broiler chicken was shown to be highly polymorphic [75–77] and some SNPs are associated with traits of economic interest (see Table 1). In Pecking ducks, polymorphisms within the 5′ regulatory region of the *MSTN* gene were associated with muscle and fat deposition [78], corroborating to the known roles played by this gene on both myogenesis and adipogenesis.

## Fish *mstn* gene promoter

In teleost fishes the study of *mstn* genes is much more complex, since this group of animals underwent an additional round of whole genome duplication in comparison to other jawed vertebrates [79]. Thus, teleost fish genomes hold two *mstn* genes: *mstn-1* (*mstn b*, *gdf8*) and *mstn-2* (*mstn a*, *gdf8I*) and even up to four copies in salmonids, because of an additional duplication in this group [80].

In the model-organism zebrafish (*Danio rerio*), Xu et al. [81] identified seven putative E-boxes in 1.2 kb of the *mstn-1* 5′ regulatory region (Fig. 1), some of which are similar in position to the ones observed in bovine, indicating a possible conserved role of these E-boxes in regulating promoter activity. A construct containing this 5′ regulatory region upstream of the GFP was injected in early-staged embryos and directed reporter gene activity in muscle cells, demonstrating that the analysed region contains muscle-specific regulatory elements [81]. However, reporter gene expression was also observed in other tissues, namely in the brain, which corroborates the hypothesis that *mstn-1* is also involved in other processes besides myogenesis [81, 82]. Many putative TFBSs were identified in *mstn-1* and *mstn-2* promoters, including TATA boxes, E-boxes (Fig. 1), MEF2 and MEF3 sites among others (Additional file 1).

In the marine fish *Sparus aurata*, 1.4 kb of the *mstn-1* 5′ regulatory region were analysed and revealed the presence of ten E-boxes arranged in three clusters (Fig. 1), besides several putative TFBSs, including CCAAT and TATA boxes [83] (Additional file 1). The positions of a TATA box, CAAT box, GRE and CRE close to the TATA box, one POU1F1a (PIT1a) site and a GH-CSE were shown to be conserved with other fish orders. In vitro analysis showed that different deletion constructs of *mstn-1* promoter were able to drive luciferase activity, with the highest activity obtained by the 1113 bp long fragment, suggesting the presence of repressive elements upstream of this region [83]. In regard to *Sparus aurata mstn-2*, three different promoter alleles (*mstn-2a*, *mstn-2b* and *mstn-2c*) were identified, as well as several SNPs [84]. All three alleles were shown to have high sequence identity in the most proximal 1050 bp, while divergence was observed distally. Phylogenetic analysis revealed that *mstn-2a* and *mstn-2b* are more closely related than *mstn-2c* [85]. As for populational frequency, different rates were observed, such that *mstn-2b* was shown to be more frequent, followed by *mstn-2c*, while *mstn-2a* was rather rare and found only in a heterozygous state [84]. The search for TFBSs showed the presence of potential sites for TATA binding protein and a CAAT box in the proximal promoter, among others [85] (see Additional file 1). The three variants present five conserved E-boxes in the proximal region, and additional E-boxes were identified in allele *mstn-2a* (five extra sites) and *mstn-2b* (six extra sites). A shorter variant of allele *mstn-2a*, lacking two E-boxes, was also identified. All three promoter alleles were tested for their activities, through reporter assays using both muscle and non-muscle cells, and were shown to be activated in both, although at a higher rate in the first ones, especially upon differentiation. Interestingly, promoter activity was upregulated upon neural cells (PC12) differentiation, which is in agreement *mstn-2* expression in brain. Deletion experiments have shown that the core promoter is contained within the most proximal 127 bp upstream of the ATG site, which include an atypical TATA sequence and a CCAAT box, as well as a site for SP1 and the initiator element (INR). *In vivo* experiments using zebrafish embryo injection of GFP reporter constructs directed by the different promoter alleles as well as luciferase assays of intramuscular injections confirmed the ability of these sequences to drive gene expression, in muscle and non-muscle tissues. Although all alleles functioned as promoters, allele *mstn-2b* was shown to be stronger than *mstn-2a* and *mstn-2c* in driving gene activity [85]. Knowing that *mstn-2b* allele presents a higher activity, it would be interesting to identify exactly the molecular mechanisms responsible for that difference. It is possible that there have been changes in TFBS sequences in this particular allele to allow chances in transcriptional activity.

The large yellow croaker (*Larimichthys crocea*), a fish cultivated in China, has many potential binding sites gathered in *mstn-1* and *mstn-2* promoters (Additional file 1), but more muscle related sites were identified in *mstn-1* promoter [86]. In *mstn-1* regulatory region the authors identified six E-boxes, as opposed to only one observed in the *mstn-2* 5′ regulatory region, although the size of this region (1029 bp) was almost twice the size of the one analysed in *mstn-2* (643 bp). The regulatory regions were also divergent in sequence, since while *mstn-1* promoter presented up to 90% of sequence identity when compared to sequences of other teleosts, *mstn-2* promoter region was shown to be less conserved in relation to other teleost *mstn-2*, as expected. Nevertheless, other putative sites were identified in both promoters, as shown in Additional file 1. To test the roles of these regulatory regions, a series of truncated constructs was produced, and their activity was measured using transfection of either CIK (grass carp kidney cells) or L6 (rat skeletal muscle cells) cells in vitro. These assays revealed the presence of inhibitors and enhancers in *mstn-1* and *mstn-2* promoters that work in a muscle specific manner [86].

The orange-spotted grouper (*Epinephelus coioides*) displays a TATA and CAAT boxes at positions − 18 and − 66 bp, respectively, as well as ten potential E-boxes in a fragment of approximately 1.9 kb of the 5′ region of *stn-1* [87]. Other myogenic binding sites were also identified, such as MEF2, MTBF and GFI-1B, as summarized in Additional file 1. A series of deletions were used to determine the role of each E-box, and showed that the presence of E5 significantly decreased promoter activity, as indicated by luciferase assays, both *in vitro* and *in vivo* [87]. Promoter activity was again elevated in the presence of E6, which displayed an antagonistic role to E5. MYOD was shown to be the main binder and activator of E6. *mstn-1* promoter activity was also investigated using a pEGFP-1 reporter vector which was co-transfected into grouper cells (GF-1), and was shown to be downregulated upon treatment with nodavirus, a member of the Betanodavirdae family, the causative agent of viral nervous necrosis or fish encephalitis. This might be the reason why naturally infected groupers show MSTN protein downregulation. On the other hand, infection with the nervous necrosis virus (NNV) significantly induced *mstn* promoter activity, indicating a possible role in immune response [87].

The sea perch (*Lateolabrax japonicus*) *mstn-1* gene has a putative TATA box in its 5′ regulatory region, as well as seven E-boxes [88] (Fig. 1). A sequence of approximately 1.1 kb was cloned upstream of a GFP reporter gene and tested for promoter activity in different cell types. GFP expression was observed in sea perch embryonic stem cells, but not in lymphocyte-like cells derived from the kidney nor in fibroblast-like cells from heart. The construct was also tested in vivo by injection into zebrafish embryos, and GFP expression was observed in back muscles of only two specimens 12 days after hatching [88].

In the barramundi (*Lates calcarifer*) promoter region of *mstn-1* gene, a putative TATA box is located 28 bp upstream from the transcription start site and its surrounding sequences (± 30 bp) were shown to be conserved across teleost fish [89]. Furthermore, two putative E-boxes (811 and 398 bp upstream from the ATG translation start codon) were also identified, and E1 was verified to be conserved in the same group of species that share the identical TATA box. Four SNPs were also found, suggesting higher mutation rates in the promoter region, although these alterations did not seem to change any functional sites [89].

The flatfish spotted halibut (*Verasper variegatus*) contains putative TATA and CAAT boxes, as well as three E-boxes in a 800-kb fragment of *mstn-1* upstream region. Some SNPs were identified and possibly have an effect on promoter activity (see Table 1) [90].

The shi drum (*Umbrina cirrosa*) *mstn-2* gene promoter has two putative TATA binding protein sites, one TATA box and two CCAAT boxes within 1.3 kb of its 5′ regulatory region, as well as six E-boxes and several TFBSs (see Additional file 1). Luciferase assays have shown that *mstn-2* gene promoter is active in both muscle and non-muscle cell lines, being more active in muscle cells and differentiated neural cells [91].

The Atlantic salmon (*Salmo salar*) promoter regions of *mstn-1a*, *mstn-1b* and *mstn-2a* contain several TFBSs, some of them common to specific gene orthologs and others more specific (Additional file 1) [80]. Five E-boxes were identified in the 750 bp 5′ regulatory region of salmon *mstn-1a* and the most proximal one aligned with an E-box in the *mstn-1b* promoter. Analysis of 2.4 kb of the upstream sequence of *Mstn-1b* revealed the presence of ten E-boxes. When tested, E-box number 2 was able to form complexes with homo and heterodimers of MYOD and/or E47 *in vitro*. On the other hand, paired E3-E4 were shown to strongly bind E47 homodimers and MYOD/E47 heterodimers, but binding to MYOD homodimers was very weak [80].

In brook trout (*Salvelinus fontinalis*), 500 bp upstream of *mstn-1* and *mstn-2* genes present two TATA boxes, as well as MEF2, E-boxes, and several other TFBSs (Additional file 1). In the *mstn-2* 5′ regulatory region, sites for NF-κB, androgen receptor element (ARE), and GRE were also found, and in the *mstn-1* 5′ regulatory region a SF1 site was identified. Despite the similarities in the promoters, these genes are expressed at different tissues of the brook trout [92].

In the rainbow trout (*Oncorhynchus mykiss*), the analysis of 2 kb of the gene promoter from *rtMstn-1a* and *rtMstn-1b* revealed the presence of a TATA box, several putative E-boxes (Fig. 1) and other TFBSs (Additional file 1). Some of these binding sites are specific of *rtMstn-1a* or *rtMstn-1b* [93]. Several of the same binding sites were identified in the promoter region of *rtMstn-2a* (2.4 kb) and *− 2b* (1.5 kb), including seven putative E-boxes, although differences in the TFBSs might explain the different expression patterns observed for the two genes [94].

## *mstn* gene promoter of invertebrates

The *mstn* gene promoter was already studied in some invertebrate species, revealing a remarkable structural conservation throughout evolution. For instance, in the scallop *Chlamys farreri* analysis of ~ 1.4 kb of the 5′ region of the *mstn* gene revealed the presence of several putative TFBSs, including a TATA box and muscle related factors MTBF, MEF2 and COMP, besides three E-boxes [95].

The banana shrimp *Fenneropenaeus merguiensis mstn* promoter also has TATA and CAAT boxes, as well as E-boxes and MEF2 binding sites contained in a 2-kb fragment [96]. A similar arrangement was found in 1.3 kb of the *mstn* 5′ region of the sea cucumber *Apostichopus japonicus,* where several potential binding sites were identified (see Additional file 1), including a TATA box [97].

SNPs were also already identified in some invertebrates, and the most relevant are summarized in Table 1.

## Conservation of *MSTN* gene promoter and 5′ regulatory region

**Fig. 2:**
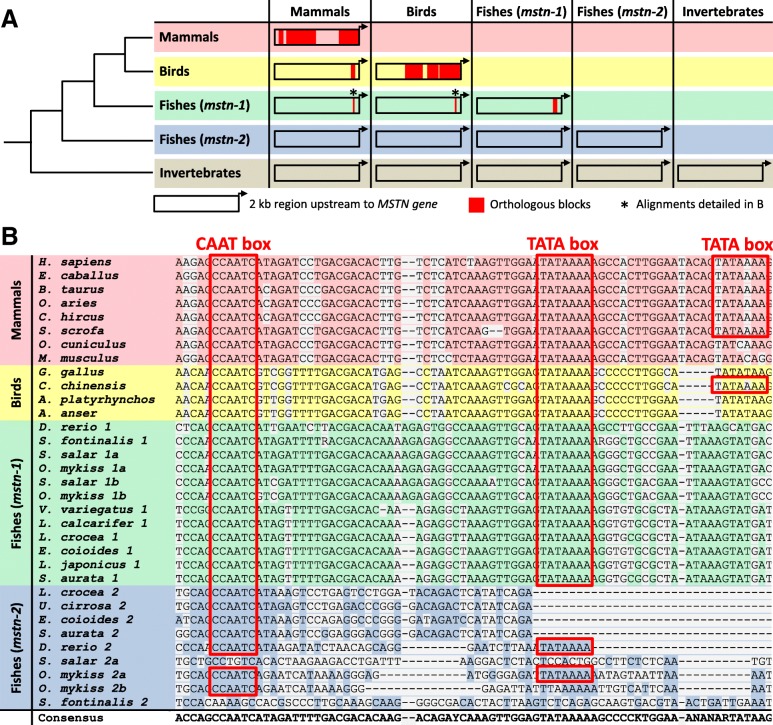
Sequence conservation of *MSTN* promoter and 5′ regulatory region across species. **a** Orthologous blocks within ~ 2 kb of *MSTN* 5′ regulatory region, based on results obtained in multiple combinations of alignments in Mulan [110], reveal evolutionarily conserved blocks mainly among species from the same group (red blocks). Only short regions of *MSTN* proximal promoter are shared by different vertebrates groups. Among fish *mstn-2* promoters, no orthologous blocks were identified when aligning the nine sequences together. Similarly, no conserved segments were found among the three invertebrate species. **b** Multiple sequence alignment of the *MSTN* proximal promoter region conserved in fishes, birds and mammals (represented by the asterisks in **a**), showing the conserved CAAT and TATA boxes (red outlined boxes). Alignment in (**b**) was obtained and adapted from MAFFT and Pro-Coffee results [111, 112]

Although the role of MSTN as an inhibitor of skeletal muscle deposition is evolutionarily conserved in animals, adjustments in the promoter and 5′ regulatory sequences may have allowed for adequacy of expression levels to species-specific demands. In fact, the literature data point to both conservation and divergence of *MSTN* promoters, depending mainly on the length of the 5′ regulatory region studied and the set of species analysed, which result in distinct conservation data [19, 25, 59]. To illustrate this, *MSTN* 5′ regulatory region fragments presented throughout this review were collected from NCBI database (accession numbers available in Additional file 1) and compared to generate alignment diagrams of 5′ regulatory regions up to ~ 2 kb, as shown in Fig. 2.

For the comparison of promoter sequences from all invertebrate and vertebrate species together, no orthologous regions of more than 100 bp and at least 70% identity were identified, since the quality of the alignment was very poor. Thus, we decided to subdivide the sequences in smaller groups for more informative comparisons, as shown in Fig. 2a.

When closely related animals were compared, the multiple alignment of *MSTN* 5′ regulatory region presented large conserved blocks, as evidenced in Fig. 2a for mammals versus mammals and birds versus birds. This high level of intra-group conservation had already been observed when different mammals were compared and several common TFBSs discovered [19]. Accordingly, many conserved regions have also been observed among birds such as chicken, duck, quail and goose [73]. However, even in related animals there are some less conserved segments within *MSTN* 5′ region, which may account for gene expression differences among these species to promote different skeletal muscle phenotypes. For instance, a phylogenetic study of the 5′ *MSTN* region indicated that ruminants form a separate cluster away from non-ruminants species (including pig and cat). Within ruminants, large species including cattle formed a separate branch from ovine and caprine [62].

When comparing different groups from the phylogenetic tree, only small orthologous blocks could be identified (Fig. 2a). In fact, the alignment of chicken *MSTN* 5′ regulatory region with mammal species (human, mouse and bovine) revealed a low level of sequence similarity (30% between chicken and human) [74], which was also observed in our analysis (Additional file 2). Similar results were obtained when comparing the 5′ regulatory region of birds with more distantly related animals, such as *Xenopus tropicalis* and zebrafish (*mstn*-*1* and *-2*), indicating sequence variation between such organisms [43]. However, when comparisons were performed with mammals and birds versus fish *mstn-1*, a short region of *MSTN* promoter was found conserved (Fig. 2a, asterisk; b). This small block of conservation was already pointed out by several authors. For instance, in the 260 bp *Mstn* proximal promoter studied by our group [25], sites for CREB and NFY, as well as a TATA box, were identified and shown to be conserved from mammals to fish. Indeed, the multiple sequence alignment of the proximal promoter sequences revealed the presence of conserved CAAT and TATA boxes is the vast majority of species analysed (Fig. 2b), which are potentially bound by NF-Y and components of the basal transcription machinery. In contrast, other binding sites identified in the proximal promoter are conserved specifically in mammals and birds, but not in fish [25], showing that promoters from birds are closer to the mammalian sequence, as they are phylogenetically closer than teleost fish promoters.

According to phylogenetic analysis, the closest ortholog of tetrapod *mstn* in teleosts fish is the *mstn-1* gene [25]. Thus, the promoter and 5′ regulatory region of fish *mstn-1* gene would be expected to display higher levels of conservation with other species than *mstn-2*. This can be observed in Fig. 2a (asterisk), although this conservation is restricted to a small block of 100 bp when comparing fish *Mstn-1* promoter to mammals or birds. Accordingly, Chen et al. [87] analysed the *mstn-1* promoter region from orange-spotted grouper (*Epinephelus coioides*) and found that it was homologous to genomic sequences of bovine, sheep and zebrafish *mstn* genes, although the authors do not present information about the size and level of conservation among the promoters of the species compared. However, Funkenstein et al. [83] observed an overall low degree of homology comparing the promoter region of *mstn-1* of six different fish orders, indicating that the sequences of teleost fish are in general more divergent. In agreement, among the 12 fish *mstn-1* promoter sequences analysed in this review, there is just a small conserved block of 140 bp for fish *mstn-1*, indicating a higher variability of the 5′ regulatory region of fishes. Sequence identity was already shown to be higher among the orders Perciformes and Pleuronectiformes, especially in the ~ 400 bp proximal promoter [83], where specific binding sites such as TATA and CAAT boxes are conserved, as is observed in our Fig. 2b. In general, *mstn-1* promoters from Salmonids (Protacanthopterygii) are more conserved among themselves than to other fishes, so in phylogenetic analyses they are grouped together, as are the members from Ostariophysi and Acanthopterygii groups [83].

In turn, the *mstn-2* gene promoter region is less conserved among the fish species included in our sample (Fig. 2a and b). In agreement, Xue et al. [86] have shown that a higher degree of conservation is observed among *mstn-1* promoter regions of teleosts than *mstn-2*, suggesting that the latter was more susceptible to variability. Only closely-related species of teleosts presented conserved blocks of sequences throughout *mstn-2* promoter region, such as the salmonids (Additional file 2). Noticeably, phylogenetic analyses of the *mstn-2* promoter have also indicated the presence of conserved CAAT and TATA boxes among Perciformes [91] such as *Larimichthys crocea, Umbrina cirrosa, Sparus aurata* and *Epinephelus coioides*, in accordance with our multiple alignment (Fig. 2b). The CAAT box of Perciformes *mstn-2* promoter was present in those species, but not the TATA box (Fig. 2b), which still may be present in other regions.

Among invertebrates, the lack of *mstn* promoter conservation (Fig. 2a) is explained by the fact that they belong to very distant groups (mollusk, echinoderm and arthropod), indicating the need for specific analyses for more precise conclusions about its conservation. We did not include the invertebrates in our alignment as they would interfere in the quality of the alignment of the other species.

## Conservation of TFBS in *MSTN* promoters

**Fig. 3:**
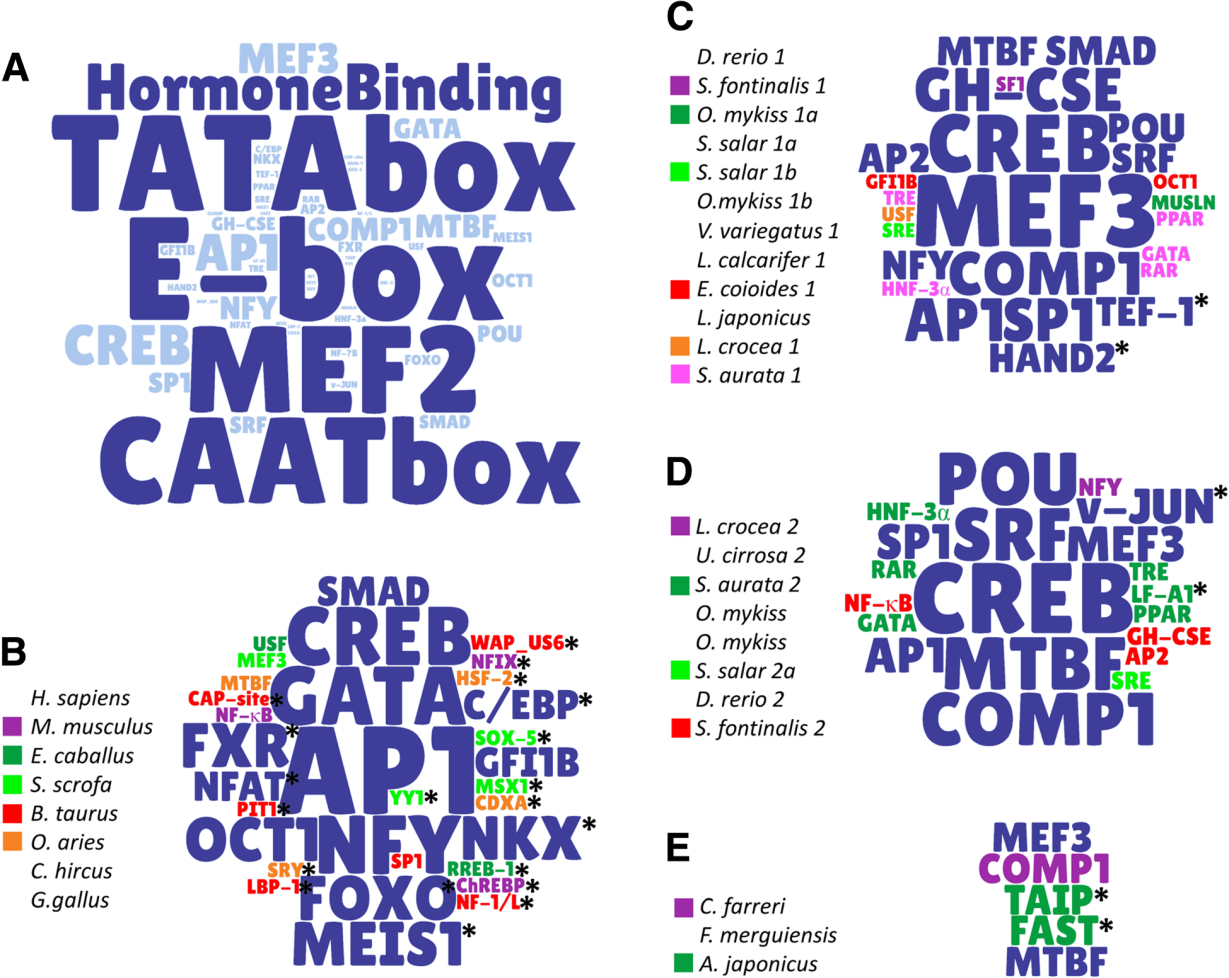
Frequency of TFBSs found in *mstn* promoter. Word cloud of the main TFBSs found in *mstn* promoter, in which letter size is directly proportional to binding site frequency among the species analysed. **a** TFBS word cloud summarizing the main TFBSs found in all species. The most frequent TFBSs (E-box, TATA box, CAAT box, MEF2 and Hormone Binding Sites) were excluded from the subsequent analyses to allow better visualization of the other sites. **b**-**e** TFBS word cloud for particular clades; **b** Amniotes; **c** Teleost fishes (*mstn-1*). **d** Teleost fishes (*mstn-2*). **e** Invertebrates. TFBSs that were exclusively identified in a specific clade are marked with an asterisk (*)

From the data above it can be seen that the promoter region of *MSTN* is conserved in some aspects among different animal groups, in particular in relation to the composition of basal and muscle-related TFBSs, as well as hormone responsiveness. Figure 3 depicts all the binding sites mentioned in this review, indicating the most common sites in larger letters. It is possible to observe that *MSTN* promoter activity is always under the influence of E-boxes, which appear in every group/species described, both vertebrates and invertebrates. Although they appear in different numbers and configurations in all analysed species, in general E-boxes exert a positive effect on *MSTN* promoter, even though some seem to be more important and preferably bound by its regulators than others, as was shown in some species. Additionally, it appears that higher numbers of E-boxes represent stronger promoter activities, although in some species it was shown that some E-boxes are sufficient to produce the same levels of activity as all E-boxes together. TATA boxes are not mentioned in only three situation (*L. crocea mstn-2; S. salar mstn-2a; O. mykiss mstn-2b*), but are present in all the other animals, almost every time accompanied by a CAAT box. Another binding site present in nearly all 5′ regions analysed is MEF2, and the combination TATA+CAAT+MEF2 + E-boxes is present in 68% (21/31) of the studied promoters (including all tetrapods), indicating a highly conserved basic structure. Since the methods used by the different authors varied, it is possible to speculate that this common core is present in all promoters, but some sites were not detected by the conditions/softwares applied. For instance, CAAT boxes were not identified in the articles discussed above in *D. rerio mstn-2*, *O. mykiss mstn-1a* and *-1b*, *L. calcarifer mstn-1* and *A. japonicus*, however further analysis performed by our group were able to localize these sites (Fig. 2b). It is also possible that some species have lost these sites in evolutionary processes. Another common trait identified in several analysed species is the responsiveness to hormones, reported in almost half (14/31) of the promoters, a feature of particular interest to the medical field.

## Diversification of *MSTN* promoter activity

The most common TFBSs among animals are essential for the basal functioning and responsiveness of the *MSTN* gene promoter, however there are also important divergences across the promoter of different species that may explain the complexity of *MSTN* gene expression in different species or cellular contexts.

For instance, the promoter of mouse and human were shown to be 25% and 300% more active in myoblasts and myotubes, respectively, than the one from cow. The responsiveness to FOXO, a known activator of the *MSTN* promoter [98], also seems to be greater in mouse when compared to cow, although the studies were carried out in mice myoblasts and might still need refinement to establish if the results are not due to a lack of bovine-specific factors absent in cell culture.

Accordingly, alignment of human, mouse, cow, rat, pig, goat and sheep promoter regions revealed the presence of five sequences strongly conserved among the large animals (human, cow, pig, goat and sheep) but modified in the small ones (mouse and rat): an additional TATA box, a CACCC motif, two A/T rich sequences and a palindrome-like sequence (PAL). Allen and Du [98] altered these five regions, with the intention of modifying the mouse sequences into the large animals sequences and observed that the alterations in the additional TATA box, the CACCC motif and the first A/T rich element reduced promoter activity. On the other hand, modifications in the second A/T rich region and in the PAL sequence caused increase in the activity, indicating that the promoter activity of the mouse *Mstn* can be modulated by converting it into the sequences observed in large species.

It is also interesting to note that the *MSTN* promoter works in association with other regulatory regions to provide the appropriate expression output. This was observed, for instance, in cattle, where two different breeds (Qinchuan and Red Angus) with different muscle phenotypes, present very similar promoters (99% homology) with comparable activity levels, indicating the necessity of other regulatory elements to adjust gene activity [99].

## *MSTN* gene promoter and epigenetics

In addition to the variety of possibilities for transcriptional regulation of the *MSTN* gene through variations in TFBSs, some studies have also investigated the role of epigenetics on the *MSTN* promoter activity. For instance, in the mouse the *Mstn* promoter was shown to be induced by treatment of Trichostatin A (TSA), a histone deacetylase inhibitor. Such inhibitors facilitate histone acetylation, causing the unwinding of packed chromatin and activation of genes [100]. This promoter induction was shown to be, at least in part, through ASK1-MKK3/6-p38 MAPK and ASK1-MKK4-JNK signaling pathways.

Moreover, maternal dietary also caused alterations in histone modifications on the *MSTN* promoter in the skeletal muscle of pigs at finishing stage [50]. A maternal low protein diet resulted in increased H3 acetylation, histone H3 lysine 27 trimethylation (H3K27me3) and decreased histone H3 lysine 9 monomethylation (H3K9me1) on the *MSTN* promoter [50]. Two gene activation markers, histone H3 lysine 4 trimethylation (H3K4me3) and H3 lysine 9 acetylation (H3K9Ac) were also observed to be significantly enriched in the *MSTN* gene promoter in low protein diet piglets [51]. These alterations, in association with others, are possibly responsible for the upregulation of *MSTN* mRNA levels observed in such piglets. On the other hand, the porcine *MSTN* promoter activity was shown to be downregulated by sulforaphane, an inhibitor of histone deacetylases, and this hypoacetylation seemed to diminish binding of MYOD to an E-box, thus causing inactivation of *Mstn* transcription [20].

In contrast, when studying the *mstn* promoter of sea perch (*Lateolabrax japonicus*), Abbas et al. [101] have seen that the methylation system is most likely not used for tissue-specific regulation of gene expression, although several CpG islands were identified in conserved regions among fish species. Analysis of sensitivity of the promoter to MNase, an endo-exonuclease that preferably digests naked DNA, showed that DNA from eye and brain, two tissues where *mstn* is expressed in this species, was highly susceptible to digestion, whereas less susceptibility was observed for heart, and even less for kidney and liver tissues, where no *mstn* expression is observed, suggesting the promoter region/gene are compacted into heterochromatin in such tissues [101].

## Potential applications

**Fig. 4:**
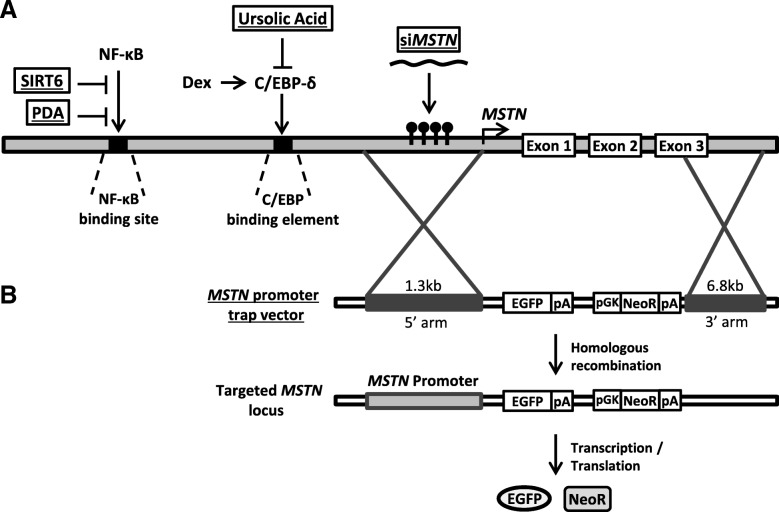
Targeting *MSTN* promoter. Schematic representation of potential mechanisms to modulate *MSTN* promoter activity for medical and breeding purposes. **a** The use of SIRT6 [103], PDA [36], ursolic Acid [105] and a specific promoter siRNA [104] were suggested to downregulate *MSTN* promoter activity to ameliorate muscle wasting; **b** Promoter trap vector was used to produce bovine cells with GFP/Neomycin genes downstream of the *MSTN* promoter, in place of the *MSTN* gene [107]

Because of its potential applications both in medicine and livestock, the mechanisms to manipulate MSTN have been extensively studied. The gene promoter itself has been target to some investigation, as described below and summarized in Fig. 4.

Regarding medical applications, it was suggested by Nogalska et al. [102] that the *MSTN* gene promoter might be activated by an increase in NF-κB levels caused by Sporadic-inclusion body myositis (s-IBM), a common disease that causes progressive muscle waste and weakness. Since MSTN levels are increased in muscle fibers suffering from s-IBM, and considering the NF-κB binding sites are found in the *MSTN* promoter region [36, 92], the authors hypothesised that interfering with this mechanism might provide a therapeutic possibility for s-IBM as well as other muscle atrophy conditions. Additionally, Samant et al. [103] have shown that SIRT6, a chromatin-bound member of the sirtuin family, is capable of suppressing *MSTN* activity by binding to NF-kB binding sites in the promoter region under normal physiological conditions, preventing the activation promoted by NF-kB binding. This suggests that restoration of SIRT6 expression may function as a therapeutic strategy to alleviate muscle wasting processes associated with chronic diseases, such as cachexia. Furthermore, pharmacologic inhibition of NF-κB by pyrrolidine dithiocarbamic acid (PDA) abolished *MSTN* promoter upregulation in response to ammonium acetate and increased myotube diameter in vitro, indicating the important role *Mstn* promoter has on sarcopenia caused by hyperammonemia in cirrhosis and other hepatic malfunctions [36].

Several methods have already been shown to decrease *MSTN* activity by interfering with its promoter. For instance, *MSTN* expression can be downregulated up to 50% with the use of siRNAs complementary to a promoter-associated transcript in C2C12 cells in culture [104]. Further analysis indicated that this regulation is mediated by histone acetylation, in an epigenetic manner, and the authors suggest this might be a new therapeutic strategy in the treatment of muscle wasting disorders.

*MSTN* expression was also shown to be downregulated via decrease in its promoter activity by ursolic acid, a ubiquitous plant compound used in the treatment of muscle wasting conditions [105]. It was reported that treatment of C2C12 myotubes with ursolic acid significantly decreased MSTN levels, through the inhibition of *MSTN* promoter activity. Furthermore, this compound was able to suppress the *MSTN* promoter overactivity normally caused by dexamethasone [31]. This suppression was shown to be mediated by a decrease in C/EBP-δ, which is normally upregulated in the presence of dexamethasone, binds to *MSTN* promoter, upregulating its activity. Upon ursolic acid treatment, C/EBP-δ levels were decreased, failing to overregulate *MSTN* promoter activity in the presence of dexamethasone. This allows improvement of overall muscle physiology, making ursolic acid a potential aid in the treatment of cachexia that accompanies several diseases [105]. Furthermore, the increase in *MSTN* promoter activity in cells exposed to dexamethasone was partially blocked by addition of glutamine, a conditionally essential amino acid during catabolic states [106]. Glutamine might act by modifying the promoter response to glucocorticoids, in a direct or indirect manner, which might shed some light into the prevention of muscle atrophy caused by glucocorticoid treatments [106].

Targeting a breeding perspective, a promoter trap vector was also shown to knockout *MSTN* in a more efficient manner than zinc finger nucleases targeting *MSTN* exons in bovine cells [107]. The promoter trap replaced the *MSTN* exons by eGFP and neomycin genes, both driven by the *MSTN* promoter, and cells were shown to be healthy and could possibly be employed in somatic cell nuclear transfer. The use of this and other genome editing technologies such as CRISPR-Cas9 may allow the modification of specific TFBSs or the number of E-boxes, which are known for their importance in modulating *MSTN* promoter activity. By targeting *MSTN* promoter, minor adjustments in the skeletal muscle phenotype of animals will become possible, and will enable to meet specific demands for improvement of animal production.

## Conclusions

Compiling the data mentioned in this review, it is possible to see that the *MSTN* gene promoter and 5′ regulatory region have both conserved and variable regions and that conservation parameters vary depending on the size and regions compared. It is important to notice that different authors have used various fragment sizes and different approaches for their analysis, resulting sometimes in conflicting data. For instance, our group have analysed a 260 bp *MSTN* promoter and concluded that, between chicken and human, there is 75% of sequence identity [25], while Gu et al. [73] found only 30% of homology between the promoters of these species, but in this case, a much longer fragment was considered (1.2 kb).

The role of E-boxes on *MSTN* promoter is consensus. They work in an additive manner to induce promoter activity under normal conditions, although some are stronger and more important than others for the performance of the promoter. Together with TATA and CAAT boxes, they compose the core of this regulatory region in the majority of animals. MEF2 completes the scenario for all tetrapods, and together, E-boxes+TATA-box+CAAT-box+MEF2 orchestrate *MSTN* promoter operation in a vast number of species. Another important feature for *MSTN* promoter, especially in mammals, is the ability to respond to hormones, and this should be taken into consideration when conditions and/or treatments that involve alterations or the use of hormones are involved.

In several different species, SNPs in the promoter region were shown to be related to body structure, indicating that this is a region of potential interest for analysis and possible interference, especially for breeding purposes. In fact, recent studies have been demonstrating potential manners to interfere with *MSTN* by targeting its gene promoter for both therapeutical and breeding purposes, indicating the great importance of studying the mechanisms underlying *MSTN* transcription. It is noticeable, however, that most studies aimed at identifying SNPs usually target the identification of markers for animal selection, and do not pursue functional analysis to understand what exactly these alterations cause in the dynamics of the gene promoter, leaving gaps in the whole comprehension of *MSTN* gene regulation.

Lastly, it is relevant to mention that besides the promoter, several other regulators are also important for the proper functioning of *MSTN* gene and protein. The role of several transcription factors [28], epigenetic regulators, post-transcriptional [14], and post-translational [9, 108, 109] controllers are slowly being unravelled and it is becoming clear that a complex network of elements is necessary to determine the moment and place MSTN should act in several physiological contexts.

## Additional files

Additional file 1: **Table S1.** Summarized information on the different *MSTN* promoters analysed in this review. Information includes accession numbers, number of E-boxes and presence (x) or absence of TFBS in each species. TFBS or sequences identified independently by our group are indicated by a red asterisk (*) [119]. (XLS 239 kb)

Additional file 2: **Table S2.** Percent identity matrix of *MSTN* promoter sequences for all the vertebrate species analysed in this review. Multiple sequence alignment was used to calculate the % id matrix in BioEdit [113]. (XLS 189 kb)

## Abbreviations

ANGII: Angiotensin II
AP-1: Activator protein 1
ARE: Androgen receptor element
C/EBPα: CCAAT/enhancer-binding protein-α
ChoRE: Carbohydrate responsive element
ChREBP: Carbohydrate responsive element binding protein
CIK: Grass carp kidney cells
COMP1: Cooperates with myogenic proteins 1
CREB: CRE binding protein
ERE-1: Equine repetitive element 1
FAST-1: Forkhead activin signal transducer
GFI-1B: Growth factor independence 1 zinc finger protein
GH-CSE: Growth hormone cell specific element
GH-CSE2: Growth hormone-cell specific element
GR: Glucocorticoid receptor
GRE: Glucocorticoid responsive element
HAND2: Heart, autonomic nervous system, neural crest derivative 2
IGF-I: Insulin-like growth factor I
INR: Initiator element
L6: Rat skeletal muscle cells
MEF2: Myocyte enhancer factor 2
MRFs: Myogenic Regulatory Factors
MSTN: Myostatin
MTBF: Mt binding site
MUSLN: Muscle initiator
NFAT: Nuclear factor of activated T
NFIX: Nuclear factor I/X
NF-κB: Nuclear factor-κB
NF-κB-p65: Nuclear factor kappa B – subunit 65
NNV: Nervous necrosis virus
NOR-1: Neuron-derived orphan receptor 1
OCT-1: Octamer transcription factor 1
PAL: Palindrome-like sequence
PPARγ: Proliferator-activated receptor-γ
PRE: Progesterone receptor binding site
RREB-1: RAS-responsive element binding protein 1
s-IBM: Sporadic-inclusion body myositis
SINE: Repetitive DNA sequence element
SNPs: Single nucleotide polymorphisms
SRE: Serum response element
SREBP1c: Sterol regulatory element-binding protein 1c
SREBP-1c: Sterol regulatory element-binding protein 1c
SRF: Serum response factor
TAIP: TGF-beta induced apoptosis protein
TEF-1: Transcriptional enhancer factor 1
TGF-β: Transforming growth factor beta
TSA: Trichostatin A
USF: Upstream stimulator factor
